# Puerpueral Sepsis

**Published:** 1895-08

**Authors:** 


PUERPERAL SEPSIS.—Dr. William Warren Potter, in discussing this subject (*Buffalo Medical and Surgical Reporter*), offers the following conclusions:

1. Obstetric engagements once accepted should be faithfully fulfilled, no matter how awkwardly they fit. Apply the same rule of cleanliness to rich and poor alike. Decline service when this cannot be done. Human life is too precious to jeopardize it by slipshod, half-hearted, or indifferent service.

2. The physician should be a model of cleanliness in body and clothing, and should insist upon the observance of similar conditions by all persons in and about the lying-in chamber.

3. The delivery room, whether in hovel or palace, court, alley, or avenue, should be simple in its furniture and hangings, and be cleaned with soap, water, and whitewash (if possible to use the latter) immediately before occupancy by the puerpera.

4. The delivery bed should consist of a new tick, filled with sweet and clean straw, covered with a blanket, impervious dressing, and a folded sheet, with other clean covering to be allowed, according to season. Exceptions to this simple bed should be as few as possible, and in no event should a bed be substituted that has been used by the sick, or that is not beyond even a suspicion of infection.

5. The patient should be specially prepared for delivery by baths and enemata, vaginal douches, and clean clothing; and labor should be conducted on the lines of absolute cleanliness, with a few digital examinations, and a complete delivery of the secundines.

6. Lesions of the genital tract should receive careful attention; rents of the perineum should be repaired, and so, too, in some instances, should tears of the cervix.

7. Antiseptic solutions containing a germicide should be used for cleaning the hands and instruments of the operator. Intra-uterine irrigation with sterilized water should be carefully employed after operative midwifery, either manual or instrumental.

8. Finally, if sepsis proceed to suppuration and abscess, the abdomen should be opened, pus cavities emptied, irrigation used, and drainage established. If the uterus and adnexa become thoroughly infected, they should be extirpated.

